# Efficacy and Safety of Edoxaban 15 mg According to Renal Function in Very Elderly Patients With Atrial Fibrillation: A Subanalysis of the ELDERCARE-AF Trial

**DOI:** 10.1161/CIRCULATIONAHA.121.057190

**Published:** 2022-03-01

**Authors:** Tetsuro Yoshida, Akihiro Nakamura, Junichi Funada, Mari Amino, Wataru Shimizu, Masayuki Fukuzawa, Saori Watanabe, Takuya Hayashi, Takeshi Yamashita, Ken Okumura, Masaharu Akao

**Affiliations:** Department of Cardiovascular Medicine, Onga Nakama Medical Association Onga Hospital, Japan (T. Yoshida).; Department of Cardiology, Iwate Prefectural Central Hospital, Morioka, Japan (A.N.).; Department of Cardiology, National Hospital Organization Ehime Medical Center, Toon, Japan (J.F.).; Department of Cardiology, Tokai University, Isehara, Japan (M.A.).; Department of Cardiovascular Medicine, Nippon Medical School, Tokyo, Japan (W.S.).; Cardiovascular Group, Primary Medical Science Department, Japan Business Unit (M.F.), Daiichi Sankyo Co., Ltd., Tokyo, Japan.; Clinical Development Department III, Development Function, Research and Development Division (S.W.), Daiichi Sankyo Co., Ltd., Tokyo, Japan.; The Data Intelligence Group, Data Intelligence Department, Digital Transformation Management Division (T.H.), Daiichi Sankyo Co., Ltd., Tokyo, Japan.; Department of Cardiovascular Medicine, The Cardiovascular Institute, Tokyo, Japan (T. Yamashita).; Division of Cardiology, Saiseikai Kumamoto Hospital, Kumamoto, Japan (K.O.).; Department of Cardiology, National Hospital Organization Kyoto Medical Center, Kyoto, Japan (M.A.).

**Keywords:** aged, anticoagulants, atrial fibrillation, edoxaban, hemorrhage, kidney, thromboembolism

The ELDERCARE-AF trial (The Edoxaban Low-Dose for Elder Care in AF Patients) demonstrated that a once-daily 15-mg dose of edoxaban was superior to placebo in preventing stroke or systemic embolism and did not result in a significantly higher incidence of major bleeding than placebo in very elderly Japanese patients with nonvalvular atrial fibrillation (NVAF) who were not appropriate candidates for standard doses of oral anticoagulants.^1^ In this prespecified subanalysis of the ELDERCARE-AF trial, we evaluated the association of renal function with the efficacy and safety of edoxaban 15 mg in these patients.

Eligible patients were ≥80 years of age and had a history of NVAF and a CHADS_2_ score of ≥2. Patients had to be considered ineligible for oral anticoagulants (warfarin, dabigatran, rivaroxaban, apixaban, or edoxaban) at the recommended therapeutic strength or approved dose for ≥1 of the following reasons: low creatinine clearance (CrCl; 15–30 mL/min), history of bleeding from a critical area or organ, low body weight (≤45 kg), continuous use of nonsteroidal anti-inflammatory drugs, and current use of an antiplatelet drug. The primary efficacy end point was the incidence of stroke or systemic embolism, and the primary safety end point was the International Society on Thrombosis and Haemostasis major bleeding. Other end points were all-cause mortality and net clinical outcome (the composite of stroke, systemic embolism, major bleeding, or all-cause mortality). Anonymized trial data will be made available at https://search.vivli.org/ to researchers on reasonable request to the corresponding author with approval by Daiichi Sankyo Co., Ltd.

In this trial, 984 patients were randomly assigned to treatment (edoxaban group, n=492; placebo group, n=492) and 681 completed the trial. For this analysis, patients in the ELDERCARE-AF trial were classified into 3 subgroups on the basis of their baseline renal function: severe renal dysfunction (CrCl 15 to <30 mL/min, n=401), moderate renal dysfunction (CrCl 30–50 mL/min, n=422), and normal renal function/mild renal dysfunction (CrCl >50 mL/min, n=161). The median duration of follow-up was 466.0 days (interquartile range, 293.5–708.0). Event rates for primary end points were calculated for each renal function subgroup. The cumulative incidences of stroke or systemic embolism and major bleeding were estimated using the Kaplan-Meier method. A Cox proportional hazards model was used to compare outcomes between the treatment groups, with the results expressed as a hazard ratio with a 95% confidence interval. Proportionality of hazards for the primary end point was confirmed by inspection of log-log survival plots. Interaction effects between treatment arms and CrCl subgroups were tested on the basis of a joint test using Wald statistics. The protocol was approved by the institute ethics committee. Written informed consent was obtained from all participants (or legal representatives for patients with cognitive impairment) before enrollment.

Kaplan-Meier curves and forest plots showing the risk of stroke/systemic embolism (primary efficacy end point) and major bleeding (primary safety end point) in the edoxaban and placebo groups according to baseline CrCl subgroups are shown in the Figure. The effect of edoxaban versus placebo on stroke/systemic embolism was consistent among all CrCl subgroups (*P* value for interaction=0.91). The increase in major bleeding with edoxaban was nonsignificant, and there was no heterogeneity between the CrCl groups (*P* value for interaction=0.63). Regarding all-cause mortality, the hazard ratios of the edoxaban groups were 0.97 for the CrCl 15 to <30 mL/min subgroup, 1.12 for the CrCl 30 to 50 mL/min subgroup, and 0.86 for the CrCl >50 mL/min subgroup (*P* value for interaction=0.90), and those for net clinical outcome were 0.91, 0.87, and 0.77 for each CrCl subgroup, respectively (*P* value for interaction=0.96). No heterogeneity was observed between the groups.

**Figure.**
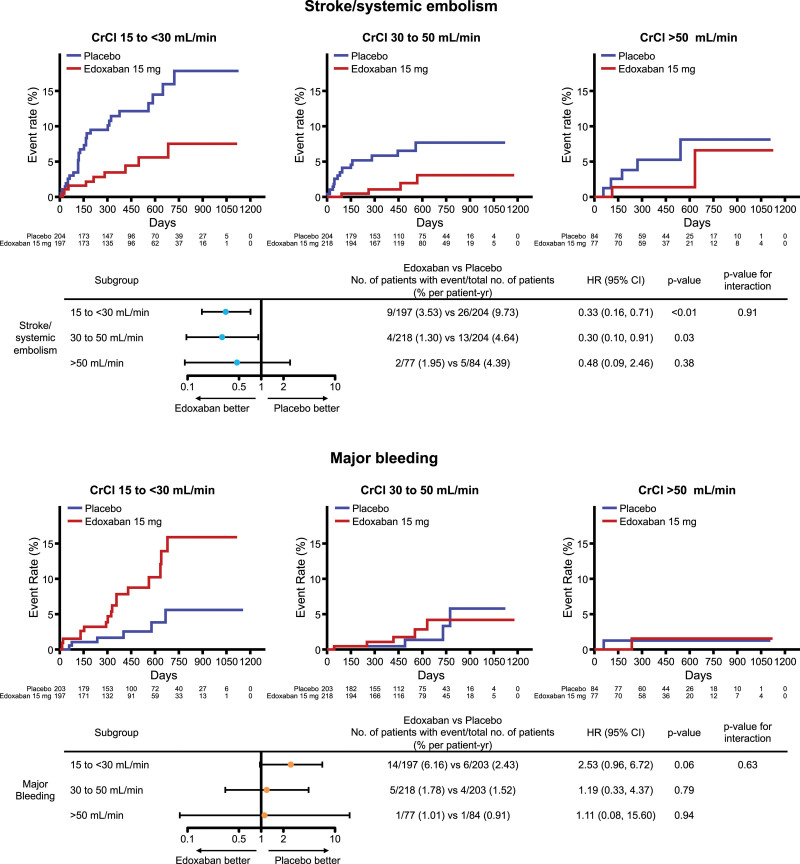
**Relationship between stroke/systemic embolism or major bleeding and renal function in patients with nonvalvular atrial fibrillation.** Kaplan-Meier curves and forest plots for stroke/systemic embolism and major bleeding in the edoxaban and placebo groups according to baseline creatinine clearance. CI indicates confidence interval; CrCl, creatinine clearance; and HR, hazard ratio.

In a subanalysis of the ENGAGE AF-TIMI 48 trial (Effective Anticoagulation With Factor Xa Next Generation in Atrial Fibrillation-Thrombolysis in Myocardial Infarction Study 48), the advantage of edoxaban over warfarin was consistent across the range of renal function in patients with NVAF (the minimum renal function was CrCl 30 mL/min).^2^ Another study showed that edoxaban 15 mg in patients with NVAF and severe renal dysfunction (CrCl 15 to <30 mL/min) exhibited safety and plasma concentration similar to the 30-mg dose in patients with normal renal function or mild dysfunction (CrCl ≥50 mL/min).^3^ In the present study, edoxaban 15 mg reduced the incidence of stroke/systemic embolism regardless of the level of renal dysfunction. There was no increase in intracranial hemorrhage or fatal bleeding events in the edoxaban group.^1^ Thus, the use of edoxaban 15 mg may be a feasible and clinically acceptable therapy even in very elderly patients with severe renal dysfunction, under appropriate measures for preventing bleeding.

In addition to the study limitations described in our previous report,^1^ the sample size of this subanalysis was small, our findings are limited to very elderly patients, and the event rates were low, which may result in indeterminate conclusions. Further studies are warranted.

In conclusion, the efficacy and safety of edoxaban 15 mg compared with placebo was broadly consistent across renal function subgroups in very elderly Japanese patients with NVAF who were not appropriate candidates for standard doses of oral anticoagulants.

## Article Information

Registration: URL: https://www.clinicaltrials.gov; Unique identifier: NCT02801669.

### Acknowledgments

We thank Michelle Belanger, MD, of Edanz (www.edanz.com) for providing medical writing support, which was funded by Daiichi Sankyo Co., Ltd. All authors meet the ICMJE’s authorship criteria.

### Sources of Funding

This study was supported by Daiichi Sankyo Co., Ltd.

### Disclosures

Dr Yoshida has received funding for research expenses (Onga Hospital) from Daiichi Sankyo Co., Ltd. Dr Shimizu has received grants from Daiichi Sankyo Co., Ltd. and Nippon Boehringer Ingelheim Co., Ltd.; and honoraria for lectures, presentations, speakers bureaus, manuscript writing, or educational events from Daiichi Sankyo Co., Ltd., Nippon Boehringer Ingelheim Co., Ltd., Bristol-Meyers Squibb, K.K., Bayer Yakuhin, Ltd., Pfizer Japan, Inc., Ono Pharmaceutical Co., Ltd., and Otsuka Pharmaceutical Co., Ltd. M. Fukuzawa, S. Watanabe, and T. Hayashi are employees at Daiichi Sankyo Co., Ltd. Dr Yamashita has received personal fees (as a medical expert) from Daiichi Sankyo Co., Ltd. for this study; and grants and lecture fees from Daiichi Sankyo Co., Ltd., Bristol-Myers Squibb K.K., and Bayer Yakuhin Ltd.; lectures fees from Pfizer Japan Inc., Nippon Boehringer Ingelheim Co., Ltd., and Ono Pharmaceutical Co., Ltd.; medical advisory fees from Toa Eiyo Ltd.; and lecture and medical advisory fees from Novartis Pharma K.K. outside the submitted work. Dr Okumura has received grants and a commission fee for the study design from Daiichi Sankyo Co., Ltd. for the submitted work; and lecture fees from Daiichi Sankyo Co., Ltd., Nippon Boehringer Ingelheim Co., Ltd., Bristol-Myers Squibb K.K., Medtronic Japan Co., Ltd., Johnson & Johnson K.K., and Bayer Yakuhin Ltd. outside the submitted work. Dr Akao has received funding support for the present manuscript from Daiichi Sankyo Co., Ltd. and grants from Bayer Yakuhin, Ltd., Pfizer Japan, Inc., Bristol-Meyers Squibb, K.K., Nippon Boehringer Ingelheim Co., Bayer Yakuhin, Ltd., and Daiichi Sankyo Co., Ltd. The other authors report no conflicts.
